# Extracellular distribution of galectin-10 in the esophageal mucosa of patients with eosinophilic esophagitis

**DOI:** 10.1093/cei/uxad026

**Published:** 2023-02-20

**Authors:** Sofie Albinsson, Christine Lingblom, Leif Johansson, Helen Larsson, Christine Wennerås

**Affiliations:** Institute of Biomedicine, Department of Infectious Diseases, Sahlgrenska Academy, University of Gothenburg, Gothenburg, Sweden; Institute of Biomedicine, Department of Infectious Diseases, Sahlgrenska Academy, University of Gothenburg, Gothenburg, Sweden; Department of Clinical Microbiology, Sahlgrenska University Hospital, Region Västra Götaland, Gothenburg, Sweden; Department of ENT, Head and Neck Surgery, Skövde County Hospital, Region Västra Götaland, Skövde, Sweden; Institute of Clinical Sciences, Department of Otorhinolaryngology, Head and Neck Surgery, Sahlgrenska Academy, University of Gothenburg, Gothenburg, Sweden; Department of Otorhinolaryngology, Head and Neck Surgery, NU-Hospital Group, Region Västra Götaland, Trollhättan, Sweden; Institute of Biomedicine, Department of Infectious Diseases, Sahlgrenska Academy, University of Gothenburg, Gothenburg, Sweden; Department of Clinical Microbiology, Sahlgrenska University Hospital, Region Västra Götaland, Gothenburg, Sweden

**Keywords:** CD16, eosinophilic esophagitis, eosinophils, extracellular vesicles, galectin-10, T cells

## Abstract

Eosinophilic esophagitis is a T-cell-driven allergic condition hallmarked by eosinophil infiltration of the esophagus. Eosinophils exposed to proliferating T cells release galectin-10 and have T-cell suppressive function *in vitro*. The aims of this study were to evaluate if eosinophils co-localize with T cells and release galectin-10 in the esophagus of patients with eosinophilic esophagitis. Esophageal biopsies from 20 patients with eosinophilic esophagitis were stained for major basic protein, galectin-10, CD4, CD8, CD16, and CD81 and analyzed by immunofluorescence confocal microscopy before and after topical corticosteroid treatment. CD4+ T-cell numbers decreased in the esophageal mucosa of responders to treatment but not in the non-responders. Suppressive (CD16+) eosinophils were present in the esophageal mucosa of patients with active disease and decreased after successful treatment. Unexpectedly, eosinophils and T cells were not in direct contact with each other. Instead, the esophageal eosinophils released large amounts of galectin-10-containing extracellular vesicles and featured cytoplasmic projections that contained galectin-10, both of which disappeared from the esophagus of the responders but remained in the non-responders. To conclude, the presence of CD16+ eosinophils together with the massive release of galectin-10-containing extracellular vesicles in the esophageal mucosa might indicate that eosinophils exert T-cell suppression in eosinophilic esophagitis.

## Teaser

CD16+ eosinophils and large amounts of extracellular vesicles containing galectin-10, a T-cell suppressive eosinophil protein, were found in the esophageal mucosa of patients with active eosinophilic esophagitis. Both the CD16+ eosinophils and the released galectin-10-containing extracellular vesicles disappeared in successfully treated patients but remained in the mucosa of the non-responders to treatment.

## Graphical Abstract

**Figure:**
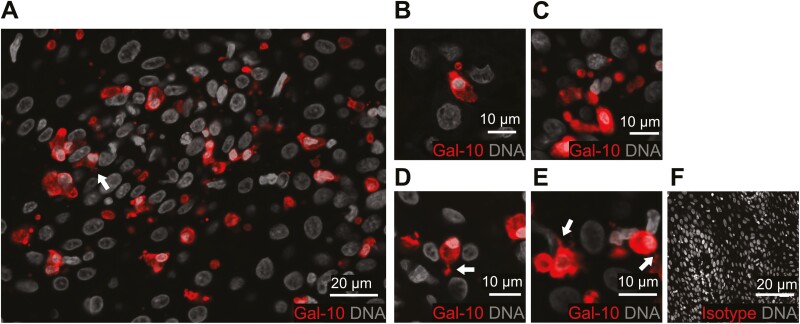

## Introduction

The esophageal disease eosinophilic esophagitis (EoE) is a chronic inflammatory condition driven by dietary or airborne allergens and the afflicted patients commonly suffer from dysphagia of solid foods [1]. Eosinophil infiltration of the esophageal mucosa is the hallmark of the disease; eosinophils are not present in the healthy esophagus [2, 3]. The mucosal levels of other immune cells, including T cells, B cells, basophils, and mast cells are also increased in EoE [4–7]. EoE is believed to be mediated by T cells; T-cell deficient mice could not be induced to develop experimental EoE [8]. Furthermore, treatment with the T-cell suppressive drug azathioprine induced clinical and histological remission in patients with EoE [9]. The CD4/CD8 T-cell ratio is lower in the inflamed esophagus compared to blood and a positive correlation of both CD4+ and CD8+ T-cell numbers with the severity of EoE has been demonstrated [5]. Although a preponderance of CD8+ T cells has been shown in the esophagus of patients with EoE [10–13], the disease appears to be strongly associated with a CD4+ Th2 inflammatory response [4]. A recent study employing single-cell RNA sequencing found a specific enrichment of CD4+ effector Th2 cells and of T regulatory cells in the esophageal mucosa of patients with EoE [5].

During the last decade, a number of studies have demonstrated that eosinophils are capable of regulating T-cell subsets and their functions [14–18]. We have identified a subgroup of eosinophils with strong T-cell suppressive properties that express CD16 on their surface [19]. Approximately 1–5% of blood eosinophils of healthy adults have surface CD16 expression, a fraction that increases when the cells are co-cultured with activated T cells [19]. We have previously shown that the fraction of these suppressive eosinophils in the blood was reduced after successful topical corticosteroid treatment of patients with EoE [20]. This population of suppressive eosinophils contains very high amounts of galectin-10, an intracellular protein that mediates the capacity of eosinophils to suppress T-cell proliferation [19].

Galectin-10 is the predominant eosinophil protein constituting approximately 10% of the cell protein mass [21–23]. It was recently reported that eosinophils experimentally induced to undergo the process of extracellular trap cell death released galectin-10 in various ways: soluble, crystallized as part of Charcot–Leyden crystals, within DNA-containing eosinophil extracellular traps and inside of plasma membrane-enveloped extracellular vesicles [24]. We have shown that eosinophils exposed *in vitro* to proliferating T cells undergo a sequential process starting with the formation of galectin-10-containing immune synapses with T cells, development of cap-like accumulations of galectin-10 on the eosinophil nuclear lobes, and ejection of the galectin-10 from the eosinophils in conjunction with nuclear DNA nets [25]. Notably, it was only the CD16-expressing eosinophils that released galectin-10 by these mechanisms [25]. The aim of this study was to explore the hypothesis that eosinophils co-localize with T cells and release galectin-10 in the esophageal mucosa to regulate the T-cell inflammation that is presumed to drive EoE.

## Materials and methods

### Patients and samples

Adult patients with EoE were recruited from the Ear Nose and Throat Department, NU Hospital Group, Trollhättan, Sweden, and Skaraborg Hospital, Skövde, Sweden for a two-part study. The first part focused on multivariate models of non-invasive biomarkers of disease and has already been published with detailed description on patient inclusion/exclusion criteria [20]. Twenty patients completed a 2-month treatment with topical corticosteroids (200 µg mometasone furoate aerosol swallowed four times per day). Esophageal biopsies and EDTA-blood (10 ml) were collected before and after treatment. The patients who exhibited histological response to treatment with <15 eosinophils/high-power field (HPF) were defined as responders to treatment and those without histological remission were defined as non-responders. Patient characteristics are listed in Table 1 and have already been published in greater detail [20]. The study was approved by the Regional Ethical Review Board of Gothenburg, Sweden (Dnr 137-09 and T664-11). Written informed consent was acquired from all study participants.

**Table 1:** Characteristics of eosinophilic esophagitis patients (*n* = 20)

Clinical data	*n*	%
*Demographic data*		
Median age (range)	43 (18–79)	—
Male	14/20	70
*Histologic findings*		
Eosinophils/HPF before treatment, median (min–max)	28 (15–80)	—
<15 eosinophils/HPF after treatment (responders)	15	75
≥15 eosinophils/HPF after treatment (non-responders)	5	25
*Endoscopic findings*		
Trachealization	15	75
Linear furrows	13	65
Plaques	11	55
Strictures	9	45
*Previous hospital interventions*		
Bolus extraction	12	60
Esophageal dilation	2	10
*Symptoms*		
Dysphagia	20	100
Chest pain	14	70
Food impaction	14	70
Cough	9	45
Nausea/vomiting	9	45
*Allergies*		
Inhalant allergy	13	65
Hay fever	8	40
Food allergy	6	30
No allergy	5	25
Food and inhalant allergy	4	20
Eczema	1	5

Abbreviation: HPF: high-power field.

### Fluorescent immunohistochemistry

Formalin-fixed paraffin-embedded esophageal biopsies cut into 4-µm sections were analyzed by immunohistochemistry. Deparaffination was done using Histo-Clear and a Tissue-Tek Linearstain II instrument (Sakura, Alphen aan den Rijn, the Netherlands). Heat-induced epitope retrieval was performed for the analysis of CD4, CD8, CD16, and major basic protein (MBP) by using a pressure cooker with EDTA Decloaker and Hot Rinse solution (Biocare Medical, Pacheco, CA, USA) followed by a wash with distilled water. Proteolytic-induced epitope retrieval was used for analysis of galectin-10 and CD81: the sections were washed with tris buffered saline (TBS) and incubated for 20 min at room temperature (RT) in a pronase solution (pronase [2 mg/ml, Sigma–Aldrich, St. Louis, MO, USA], sodium acetate [0.01 M], calcium chloride [0.005 M], TBS). Following antigen retrieval, the sections were washed with TBS, permeabilized using TBS-0.1% saponin (TBSS), incubated with blocking buffer (5% donkey serum [Sigma–Aldrich] in TBSS) for 20 min at RT, and incubated with primary antibodies (Table 2) for 1 h at RT. The tissue sections were washed with TBSS and incubated in the dark for 45 min with secondary antibodies (Table 2) and DNA stain Hoechst 34580 (Thermo Fisher Scientific, Waltham, MA, USA). The sections were washed with TBSS and TBS, mounted with ProLong Diamond Antifade Mountant (Invitrogen, Carlsbad, CA, USA) and analyzed using either a Leica TCS SP5 confocal microscope combined with the Leica Application Suite X software (Leica Microsystems, Wetzlar, Germany) or an LSM 700 confocal microscope and ZEN software (Carl Zeiss AG, Oberkochen, Germany). Negative controls consisted of isotype controls and exclusion of primary antibodies. CD16+ eosinophils and galectin-10+ eosinophils and structures were manually counted using the Cell Counter plugin of the Fiji ImageJ software [26], or the ZEN software. Eosinophils and T cells were enumerated using macros and the Cell Counter plugin of the Fiji ImageJ software. For the enumeration of eosinophils and T cells in the tissue, the area of the visible tissue in the confocal microscopy image with the peak number of eosinophils was calculated and the number of cells present within the area was converted to cells/mm^2^ to give a comparable measure between sections.

**Table 2:** Antibodies used for fluorescent immunohistochemistry

Antigen target	Clone	Isotype	Conjugate	Manufacturer
*Primary antibodies*				
CD4	pAb	Goat IgG	Unconjugated	R&D Systems
CD8	C8/144B	Mouse IgG_1_,κ	Unconjugated	Dako
CD16	DJ130c	Mouse IgG_1_	Unconjugated	Bio-Rad
CD81	SN206-01	Rabbit IgG	Unconjugated	Invitrogen
Galectin-10	B-F42	Mouse IgG1,κ	Unconjugated	Diaclone
Major basic protein	pAb	Rabbit IgG	Unconjugated	Atlas antibodies
*Secondary antibodies*				
Goat	pAb	Donkey IgG	AF555	Thermo Fisher Scientific
Mouse	pAb	Donkey IgG	AF647	Thermo Fisher Scientific
Rabbit	pAb	Donkey IgG	AF488	Thermo Fisher Scientific
*Isotype controls*				
Goat isotype control	pAb	Goat IgG	Unconjugated	Thermo Fisher Scientific
Mouse isotype control	P3.6.2.8.1	Mouse IgG1,κ	Unconjugated	Thermo Fisher Scientific
Rabbit isotype control	pAb	Rabbit IgG	Unconjugated	SouthernBiotech

### Flow cytometry analyses

Blood samples collected in conjunction with the esophageal biopsies were analyzed by flow cytometry, the method and results of which have already been published [20]. The blood CD4/CD8 T-cell ratios in the previous study were compared to the CD4/CD8 T-cell ratios in the esophageal sections in the current study.

### Statistics

Paired groups were analyzed with the Wilcoxon matched-pairs signed rank test, unpaired groups with the Mann–Whitney test, and correlation between data sets was analyzed with Spearman’s rank correlation, by using GraphPad Prism 9.1.2 software (GraphPad, San Diego, CA, USA). A *P*-value of <0.05 was considered statistically significant.

## Results

### Histological response to topical corticosteroid treatment

**Figure 1:**
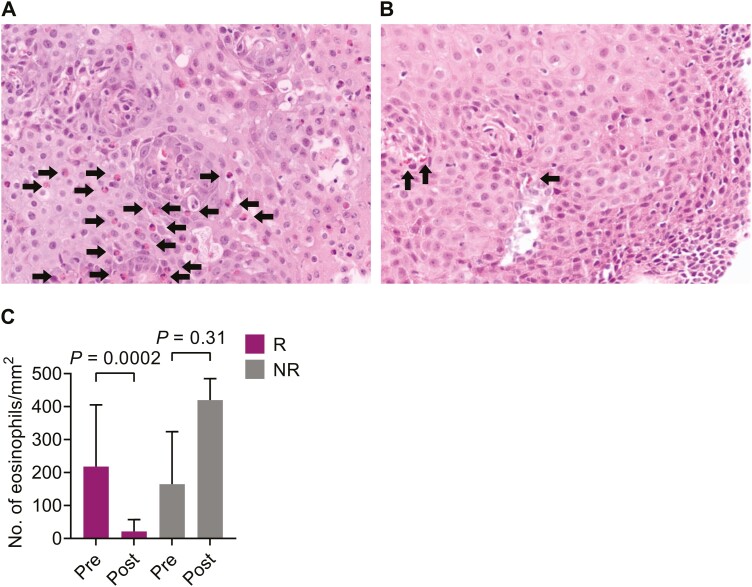
: Eosinophil numbers in the esophageal mucosa decrease after successful treatment of eosinophilic esophagitis. Hematoxylin-eosin stainings of a responder patient with eosinophilic esophagitis (**A**) before and (**B**) after treatment with topical corticosteroids. Arrows point toward eosinophils in a part of the eosinophil dense section in (A) and the eosinophils present in (B). (**C**) Comparison of number of tissue eosinophils/mm^2^ in responders (R, *n* = 15) and non-responders (NR, *n* = 5) pre-treatment and post-treatment. Wilcoxon matched-pairs signed rank test was used, bars show median with interquartile range

Fifteen patients attained histological response to treatment with <15 eosinophils/HPF (responders). An example of mucosal eosinophil infiltration before and after treatment of a responder is presented in Fig. 1A–B. Five patients who did not respond to treatment (≥15 eosinophils/HPF after treatment) were designated non-responders. A statistically significant decrease in eosinophil numbers per mm^2^ was seen for the responders after treatment but not for the non-responders (Fig. 1C).

### CD4+ T cell numbers decrease in the esophagus of responders to treatment

**Figure 2:**
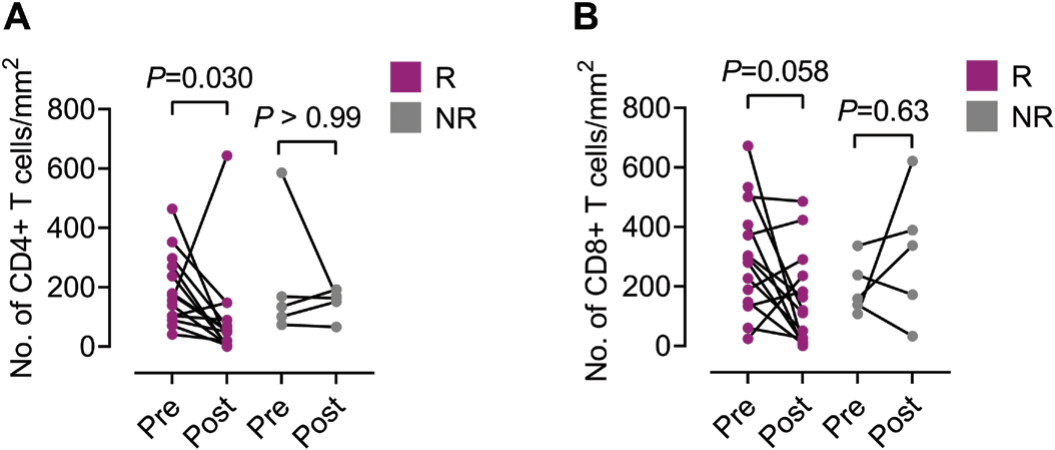
Esophageal T-cell numbers decrease in eosinophilic esophagitis patients who responded to treatment with topical corticosteroids. Comparison of number of (**A**) CD4+ and (**B**) CD8+ T cells/mm^2^ in esophageal sections with peak eosinophil numbers pre- and post-treatment for responders (R, *n* = 15) and non-responders (NR, *n* = 5). Wilcoxon matched-pairs signed rank test was used for the statistical computations

First, we examined the numbers of CD4+ and CD8+ T cells in the esophagus of patients with active EoE. Though not statistically significant (*P* = 0.087), there was a tendency for higher levels of CD8+ T cells/mm^2^ than CD4+ T cells/mm^2^ in the esophagus before treatment was initiated: a median of 238 CD8+ T cells/mm^2^ (25–75 percentile: 138–372) vs. a median of 156 CD4+ T cells/mm^2^ (25–75 percentile: 101–271). The median CD4/CD8 ratio in the esophageal mucosa was 0.63 (25–75 percentile: 0.34–1.2), which was three times lower compared to the median CD4/CD8 ratio in the blood (*P* = 0.0012). Secondly, we examined if CD4+ and CD8+ T-cell numbers in the esophagus differed between patients who responded histologically to topical steroid treatment and those who did not. No differences were found in the levels of these two types of T cells between responders and non-responders prior to treatment. In contrast, the levels of CD4+ T cells/mm^2^ decreased 3-fold in the responders but remained unchanged among the non-responders after treatment (Fig. 2A). Similarly, a 2-fold decrease was seen for the numbers of esophageal CD8+ T cells/mm^2^ among the responders, although this did not quite reach statistical significance, and the CD8+ T-cell numbers remained unchanged among the non-responders post-treatment (Fig. 2B).

### CD16+ eosinophils are present in the esophageal mucosa of patients with eosinophilic esophagitis and decrease after successful treatment

**Figure 3:**
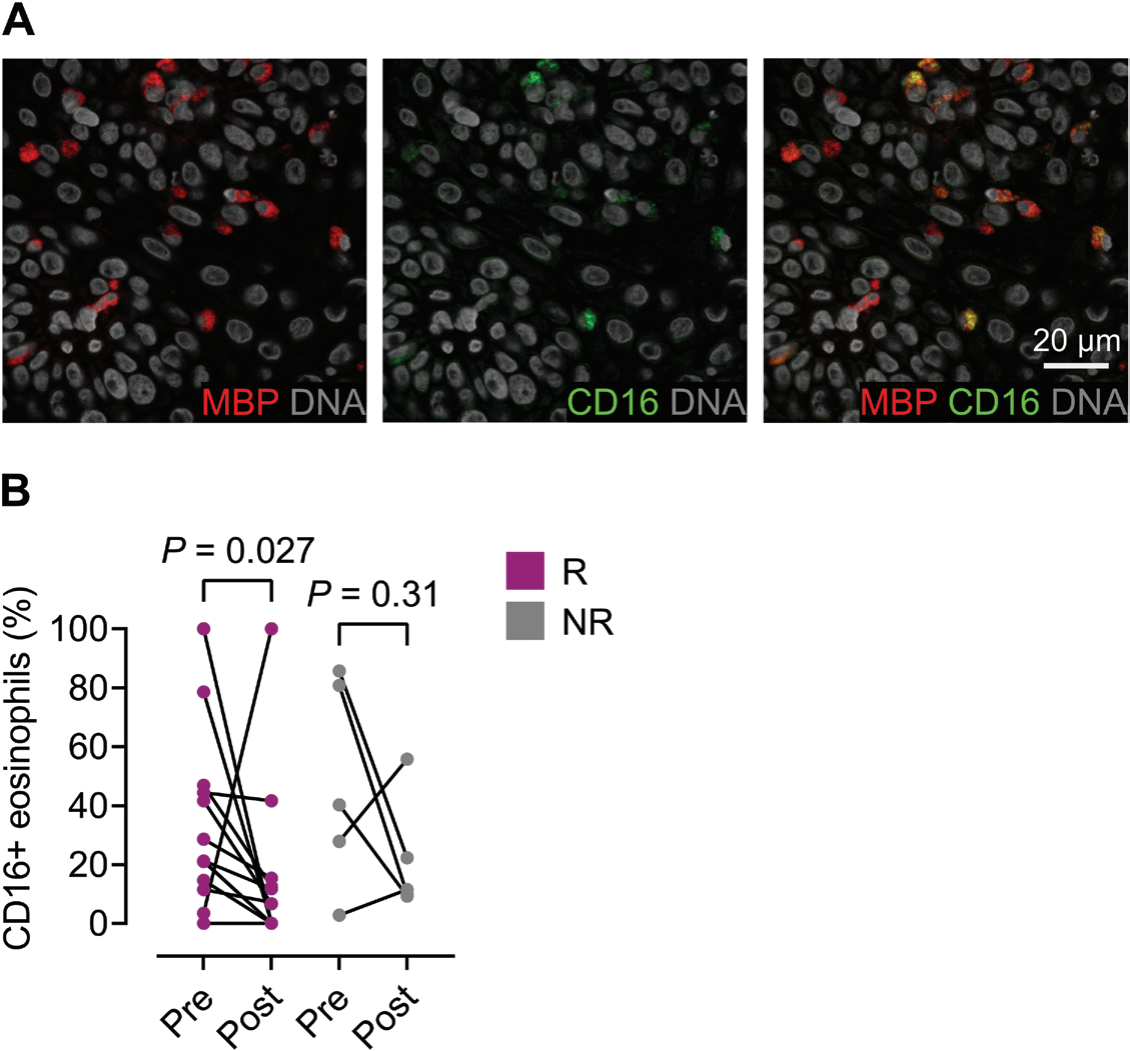
CD16-positive eosinophils are present in the esophageal mucosa of patients with eosinophilic esophagitis. (**A**) Representative fluorescent immunohistochemical staining of eosinophils (MBP) that express CD16 displayed as two separate images and as an overlay image. (**B**) Comparison of levels of tissue CD16+ eosinophils in responders (R, *n* = 15) and non-responders (NR, *n* = 5) pre-treatment and post-treatment. Wilcoxon matched-pairs signed rank test was used

Because of the superior capacity of CD16+ eosinophils to suppress T cells *in vitro* [19], we next sought to determine if the cells were present in the esophageal mucosa of patients with EoE. The biopsies were stained for the eosinophil marker MBP, CD16, and nuclear stain Hoechst 34580 (Fig. 3A), and the percentages of CD16+ eosinophils in the tissue sections with peak eosinophil numbers were determined. Before treatment, the CD16+ eosinophils constituted 28% (median, 25–75 percentile: 12–47%) of the esophageal eosinophils. After treatment, 0% (median, 25–75 percentile: 0–13%) of the eosinophils expressed CD16 among the responders. When comparing the levels of CD16+ eosinophils before and after treatment, a statistically significant decrease in the percentage of CD16+ eosinophils was evident in the responders after treatment, but not among the non-responders (Fig. 3B).

### Eosinophils are not in direct contact with T cells

**Figure 4:**
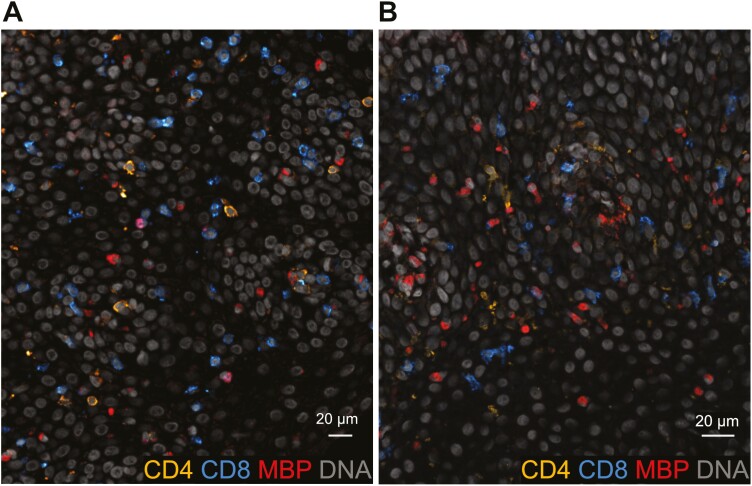
: Eosinophils are not in direct contact with T cells in esophageal sections of patients with eosinophilic esophagitis. Immunohistochemical stainings of eosinophils (MBP), CD4+ T cellss, CD8+ T cells, and nuclear DNA stain in a representative esophageal tissue section from (**A**) a patient with eosinophilic esophagitis before treatment and (**B**) in a non-responder patient after treatment

Next, we examined if eosinophils were in direct contact with CD4+ and CD8+ T cells, respectively, in the tissue sections containing peak eosinophil numbers. A mere 3.8% of the eosinophils were in direct contact with CD4+ T cells and 9.3% of the eosinophils were in direct contact with CD8+ T cells. There were no statistically significant differences regarding the fractions of eosinophils that were in direct contact with T cells either before or after treatment nor between responders and non-responders. Furthermore, visual inspection of the stained tissue sections conveyed no obvious patterns of direct cell contact (Fig. 4A and B).

### Esophageal eosinophils feature galectin-10-containing cytoplasmic projections and release extracellular vesicles containing galectin-10

**Figure 5:**
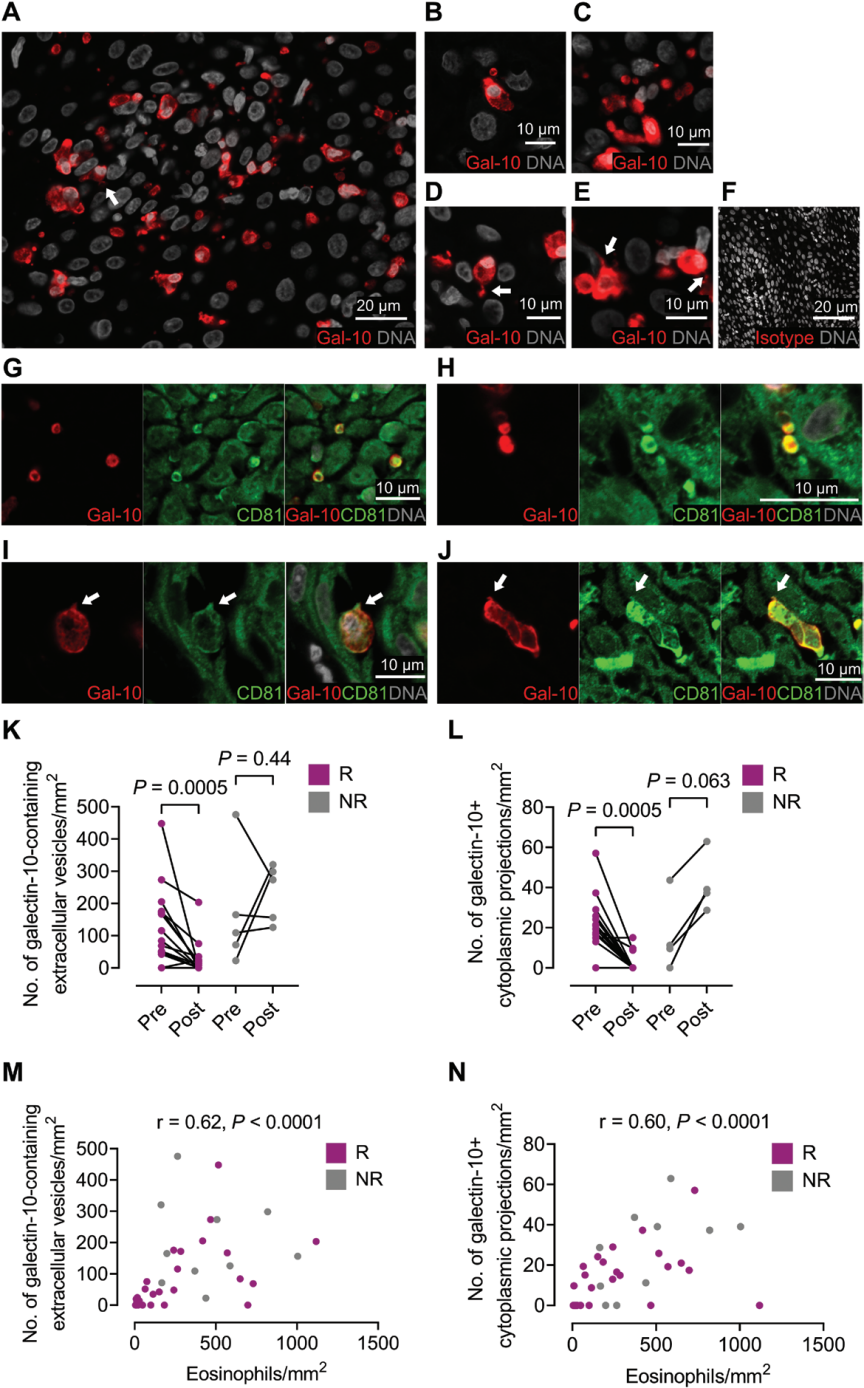
Galectin-10 is found inside of cytoplasmic projections of eosinophils and is released into the esophageal mucosa in extracellular vesicles of patients with active eosinophilic esophagitis. (**A**) Immunostaining of galectin-10 and DNA (Hoechst) in esophageal tissue from a representative patient with untreated eosinophilic esophagitis. An arrow points toward a cytoplasmic projection of galectin-10. (**B,C**) Close-up of eosinophils with adjacent extracellular vesicles containing galectin-10. (**D,E**) Magnified eosinophils with cytoplasmic projections of galectin-10. Arrows point towards the cytoplasmic projections with galectin-10. (**F**) Isotype control for galectin-10 antibody. (**G,H**) Eosinophil extracellular vesicles co-stained for CD81. (**I,J**) Eosinophil with galectin-10+ cytoplasmic projection (arrow) co-stained for CD81. (**K**) Comparison of numbers of galectin-10-containing extracellular vesicles/mm^2^ in the tissue sections with peak eosinophil numbers in responders (R, *n* = 15) and non-responders (NR, *n* = 5) pre- and post-treatment. (**L**) Univariate analysis of the number of galectin-10+ cytoplasmic projections/mm^2^ for the responders (R, *n* = 15) and non-responders (NR, *n* = 5) pre- and post-treatment. Spearman correlation between numbers of eosinophils/mm^2^ and numbers of (**M**) galectin-10-containing extracellular vesicles/mm^2^ and (**N**) galectin-10+ cytoplasmic projections/mm^2^ in the tissue sections with peak eosinophil counts of responders (R) and non-responders (NR). Wilcoxon matched-pairs signed rank test was used in (**K,L**)

Since eosinophils were not in direct contact with T cells, we investigated if galectin-10 was secreted in the esophageal mucosa of patients with active EoE. To this end, esophageal biopsies were stained for galectin-10, DNA (Hoechst), and CD81 (membrane protein), and non-cell bound galectin-10 was quantified in the tissue sections with peak eosinophil numbers. Large galectin-10+ extracellular vesicles devoid of DNA were found distributed in the tissues. Moreover, cytoplasmic projections positive for galectin-10 that originated from apparently intact eosinophils and stained positive for the membrane marker CD81 were observed. The galectin-10-containing extracellular vesicles were also positive for the membrane marker CD81 (Fig. 5A–J). These extracellular vesicles had a median diameter of 2.2 µm (25–75 percentile: 1.6–2.6 µm) and numbered a median of 109 extracellular vesicles/mm^2^ (25–75 percentile: 48–176) in the esophagus of untreated patients. After treatment, the galectin-10-containing extracellular vesicles disappeared from the esophageal mucosa of the responders but not from the non-responders whereas rather the opposite, a tendency towards increased quantities of galectin-10+ extracellular vesicles was observed (Fig. 5K). A median of 19 galectin-10+ cytoplasmic projections/mm^2^ (25–75 percentile: 11–26) were found in the esophagus before treatment, of which 12% contained visible DNA. The cytoplasmic projections containing galectin-10 also disappeared from the esophageal mucosa of the responders after treatment, and again, a tendency toward an increase was seen for the non-responders (Fig. 5L). Lastly, a strong positive correlation was seen between the number of esophageal eosinophils and the number of galectin-10+ extracellular vesicles in the mucosa, and between eosinophil numbers and the number of galectin-10+ cytoplasmic projections per mm^2^ (Fig. 5M–N). Subjects with no eosinophil numbers in the mucosa were excluded from the correlation analysis.

## Discussion

In this study, we showed that CD16+ (suppressive) eosinophils were present in the esophageal mucosa of patients with active disease and that large amounts of galectin-10-containing extracellular vesicles were released by the esophageal eosinophils. In addition, the number of esophageal CD4+ T cells, but not CD8+ T cells, was reduced following successful treatment. Wen et al. reported that only two T-cell phenotypes, regulatory T cells and CD4+ effector Th2-like cells, were enriched in the esophageal tissues of patients with EoE compared to controls and that the gene for the eosinophil stimulatory cytokine IL-5 was upregulated 1500-fold [5]. Although EoE is believed to be driven by CD4+ Th2 cells [4, 5], CD8+ T cells are more numerous than CD4+ T cells in the esophageal mucosa of patients with EoE [10, 11], which was also seen in our study. It should be pointed out that CD8+ T cells also predominate in the healthy esophagus [10]. Consistent with our findings, others have previously shown a decrease in esophageal CD3+ T cells post-corticosteroid treatment of EoE, but also a decrease of CD8+ T cells [10], a decrease which did not reach statistical significance in our study. Mouse models of EoE have demonstrated a role for CD4+ T cells, but not for CD8+ T cells in EoE pathogenesis [8].

Our findings of CD16+ eosinophils in the esophageal mucosa of patients with active EoE that decreased in the individuals who responded to treatment are novel. We have previously shown that when CD16-negative eosinophils are co-cultured with activated T cells, a subgroup of the eosinophils develop a CD16+ phenotype [19]. These CD16+ eosinophils are better at T-cell suppression than conventional eosinophils [19], make galectin-10-containing immune synapses with T cells, and release galectin-10-containing eosinophil extracellular traps (EETs) *in vitro* [25]. The presence of CD16+ eosinophils in the esophageal mucosa supported our hypothesis that one function of eosinophils might be to suppress T cells in patients with EoE. Since the majority of the eosinophils were not in direct contact with T cells in the mucosa it is unlikely that the eosinophils suppressed T cells via cell-cell contact to any large extent, contrary to our previous reports showing that cell contact was essential for eosinophil-mediated T-cell suppression, at least *in vitro* [14]. These apparently contradictory findings can be reconciled: the transwell system used to study cell contact in our previous *in vitro* study only allowed passage of nanoparticles from the eosinophil compartment to the T-cell compartment. One of the salient findings of the current study was the massive release of µm-sized, membrane-enveloped, galectin-10-containing extracellular vesicles from the esophageal eosinophils, which would have been blocked in the transwell system. Fukuchi et al. investigating a different inflammatory condition, eosinophilic granulomatosis with polyangiitis, also discovered galectin-10+ extracellular vesicles in the skin, lung, and digestive tract of patients with active disease [27].

Initially, we thought the cytoplasmic projections of galectin-10 were galectin-10-containing EETs since approximately 15% of the eosinophils in the esophageal mucosa of patients with EoE have been shown to generate EETs [28]. However, only 12% of the cytoplasmic projections of galectin-10 contained DNA. Instead, we found that the majority of the cytoplasmic projections were positive for CD81, a cell surface tetraspanin [29], making it more likely that these cytoplasmic projections are intact protrusions of the cytoplasm. In fact, galectin-10 is stored in the cytoplasm of eosinophils [30]. It is possible that some of these galectin-10+ cytoplasmic projections constitute uropods, similar to the experimentally induced galectin-10-containing uropods formed by TNF-alpha-stimulated eosinophils shown by Melo et al. using electron microscopy [30]. Another possibility is that a share of these cytoplasmic projections might arise from an early stage of local rupture of the eosinophil membrane, representing the start of cytolysis. An earlier study showed by transmission electron microscopy experiments that >80% of the eosinophils in the esophagus of patients with EoE demonstrated signs of cytolysis [31]. Moreover, release of EETs is a feature of experimentally induced cytolysis [32], which might explain the cytoplasmic projections containing galectin-10 together with DNA.

To conclude, this study presents the novel findings of suppressive CD16+ eosinophils and massive release of large galectin-10-containing extracellular vesicles in the esophageal mucosa of patients with active EoE, both of which disappeared in the mucosa of successfully treated patients. Whether this resolution was a secondary effect due to curbed T-cell inflammation or a direct effect of the topical corticosteroids on the eosinophils is presently unknown.

## Data Availability

The data underlying this article will be shared on reasonable request to the corresponding author.

## Abbreviations:

EET: eosinophil extracellular trap
EoE: eosinophilic esophagitis
HPF: high-power field
MBP: major basic protein
RT: room temperature
TBS: tris-buffered saline
TBSS: TBS-0.1 % saponin.
